# Flexible SERS Substrates for On-Site Food Safety Monitoring: A Five-Year Progress Review

**DOI:** 10.3390/foods15173070

**Published:** 2026-08-29

**Authors:** Wenzheng Ye, Zhihua Zhu, Xinran Lv, Zhizhong Sun, Zhiyu Yang, Ruiyun Zhou

**Affiliations:** 1 School of Food Science and Engineering, Jiangsu University, Zhenjiang 212013, China; 2212418049@stmail.ujs.edu.cn (W.Y.); 3230910099@stmail.ujs.edu.cn (Z.Z.); lvxinran-lxr@stmail.ujs.edu.cn (X.L.); 3240910064@stmail.ujs.edu.cn (Z.Y.); 2 College of Biological Systems Engineering and Food Science, Zhejiang University, Hangzhou 310058, China; sunzz@zju.edu.cn

**Keywords:** surface-enhanced Raman scattering, flexible substrates, food safety, on-site detection, portable spectrometer

## Abstract

Surface-enhanced Raman scattering (SERS) technology has attracted growing interest for on-site food safety monitoring, with flexible substrates offering distinct advantages in conformal sampling, portability, and non-destructive analysis. This review systematically summarizes recent advances in flexible SERS substrates for practical food safety applications, covering design strategies including material selection, structural engineering, and performance enhancement, as well as diverse applications such as swabbing-based detection, in situ analysis, enrichment-assisted trace detection, and multiplexed contaminant identification. Challenges and future perspectives are also discussed, focusing on portable instrument adaptation, simplified sample pretreatment, intelligent data analysis, and scalable manufacturing. This review aims to bridge the gap between laboratory research and field deployment, providing valuable insights for the commercialization of flexible SERS technology in real-world food safety monitoring.

## 1. Introduction

Ensuring food safety remains a persistent global challenge, driven by increasingly complex supply chains, diverse contamination sources, and strict regulatory limits on hazardous residues [1,2,3]. Conventional laboratory-based analytical techniques, such as high-performance liquid chromatography (HPLC) [4], liquid chromatography–tandem mass spectrometry (LC-MS/MS) [5], and plate-counting methods [6], offer high sensitivity and reliability. However, they are not suitable for on-site, rapid screening scenarios. Food matrices are often chemically complex and heterogeneous, containing pigments, lipids, proteins, and endogenous interferents that demand extensive sample cleanup before instrumental analysis [7,8,9]. Moreover, many high-risk samples such as fresh produce, aquatic products, bulk grains present irregular, non-planar surfaces where contaminants may be unevenly distributed, making representative sampling difficult [10]. The turnaround time of centralized lab testing often becomes a bottleneck in routine monitoring and emergency responses, including market-side residue checks, border inspections, and foodborne outbreak tracing [11,12]. As a result, there is a growing need for detection platforms that offer ultra-trace sensitivity, require minimal sample preparation, and can be operated in the field [13,14].

Surface-enhanced Raman scattering (SERS) has emerged as a promising candidate to bridge this gap. It provides molecular fingerprint specificity and high enhancement factors, typically on the order of 10^6^ to 10^8^ [15,16]. In optimized hotspot structures, it can even enable detection at the single-molecule level. For food analysis, SERS offers several key advantages: rapid acquisition within seconds, compatibility with handheld Raman devices, and the capability to detect small molecules and microbial signatures without heavy sample preparation [17,18,19]. However, conventional SERS substrates are predominantly rigid. Common examples include silicon, glass, and quartz-supported nanostructures. Such rigid substrates face limitations when the sampling target is curved, rough, or deformable, such as fruit skins, meat surfaces, and leaf textures [20,21]. Rigid tips also struggle to achieve conformal contact, leading to poor analyte transfer and spot-to-spot irreproducibility [22,23]. Over the past decade, flexible SERS substrates built on materials such as polydimethylsiloxane (PDMS) [24,25], polyethylene terephthalate (PET) [26,27], polyvinylidene fluoride (PVDF) [28,29], cellulose-derived papers [30,31], track-etched membranes [32], and electrospun mats [33] have gained traction. This is because they can adapt to surface topography, work with swabbing or immersion sampling, and in some cases integrate enrichment, separation, and detection into one step [34,35]. The shift to flexible substrates thus moves SERS from a mostly lab-confined technique toward one that can be used for in situ, on-site food safety testing.

Research on flexible SERS substrates for food applications has expanded rapidly in the last five years, with representative demonstrations spanning three major hazard categories [36,37,38]. For pesticide residue surveillance, flexible formats such as nanoparticle-decorated polymer films, Au- or Ag-loaded cellulose membranes, and magnetic metal–organic frameworks (MOF)-composite patches have been applied to solid-surface swabbing of fruits and vegetables and to liquid-phase immersion in tea, milk, and juice [39,40,41]. In the domain of foodborne pathogens, flexible SERS platforms increasingly incorporate capture-enrichment strategies [42,43]. These strategies include magnetic beads, aptamers, antibodies, and lectin functionalization [44]. When coupled with label-free bacterial fingerprinting or Raman reporter tags, such platforms were able to detect target bacteria in complex food matrices with a limit of detection (LOD) of a few CFU mL^−1^ [45,46,47]. Mycotoxin detection has also seen flexible SERS applications, including G-quadruplex DNAzyme multimodal sensors, targeting aflatoxin B_1_, zearalenone, ochratoxin A [48], and deoxynivalenol [49] in cereals, nuts, and spices [50,51,52,53]. While several review articles have summarized SERS for food analysis [54,55,56], fewer have specifically traced the recent evolution of flexible substrate engineering in connection with field-oriented detection strategies, and a focused five-year progress update that links substrate design, multi-hazard coverage, and on-site integration remains useful for guiding next-step developments.

**Figure 1:**
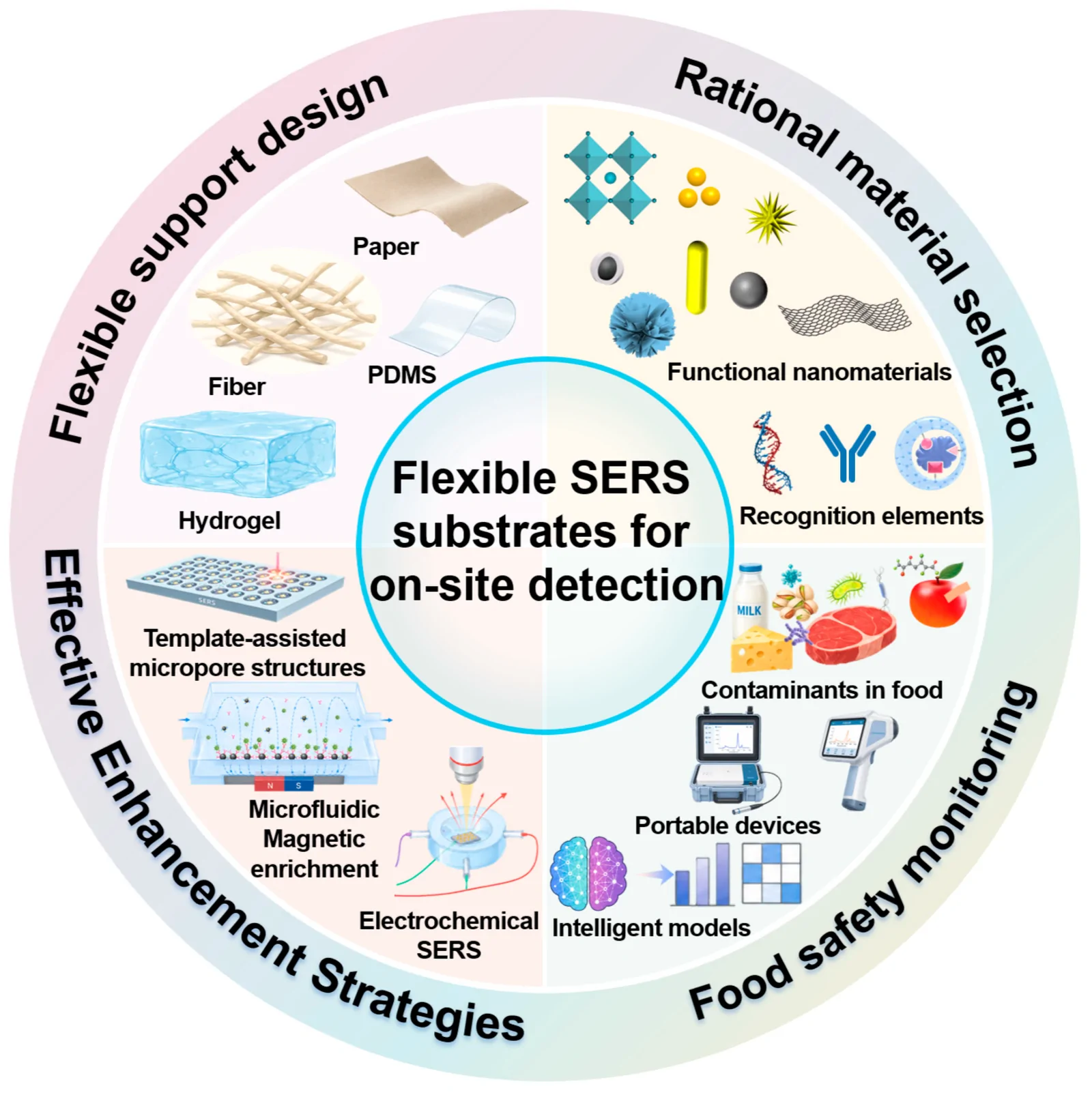
Schematic illustration of flexible SERS substrates for on-site food safety monitoring.

Given the rapid progress of flexible SERS substrates for on-site food safety monitoring, several reviews have emerged, each focusing on distinct aspects [57,58,59]. Wang et al. provided a comprehensive overview of the principles, design strategies, and diversified material selections of flexible SERS substrates [60], primarily from the perspective of substrate fabrication and fundamental mechanisms. Guo et al. discussed SERS-based on-site sensing strategies from the workflow of target signal collection, amplification, and readout [61], covering broad application scenes but not specifically dedicated to flexible substrates. Meng et al. highlighted the integration of microfluidic analytical devices with SERS for on-site food safety [62], while Si et al. emphasized artificial intelligence-assisted SERS data analysis [63]. However, these reviews typically concentrate on individual technical dimensions, including substrate design, sampling methods, microfluidic integration, or data analytics, without systematically bridging the gap from laboratory prototypes to field-deployable food safety monitoring systems. Therefore, this paper aims to underscore the translational pathway of flexible SERS technology for practical food safety applications by systematically summarizing recent advances (Figure 1): (1) design strategies for flexible SERS substrates, encompassing material selection, structural engineering, and performance enhancement; (2) diverse applications in food safety monitoring, including swabbing-based detection on irregular surfaces, in situ analysis, enrichment-assisted trace detection, and multiplexed analysis; (3) challenges and outlook, focusing on the adaptation to portable instruments, simplification of sample pretreatment, intelligent data analysis, and scalable manufacturing. By addressing these critical aspects, this review seeks to bridge the gap between laboratory research and practical deployment, thereby accelerating the commercialization of flexible SERS technology for real-world food safety monitoring.

## 2. Design Strategies for Flexible SERS Substrates

The performance of a flexible SERS substrate depends critically on its design and fabrication [64]. Over the past five years, researchers have explored a wide range of material combinations and structural configurations to improve sensitivity, uniformity, and practical usability [65,66,67]. These efforts can be grouped around three key considerations: the selection of plasmonic materials and core–shell structures, the choice of flexible supporting materials, and the optimization techniques used to enhance hotspot density, self-cleaning ability, and environmental safety.

### 2.1. Material Selection and Structural Design

Material selection directly determines the SERS enhancement capability and the adaptability of a flexible substrate to real sample surfaces [68,69,70]. A plasmonic silver-nanoparticle-reinforced poly(N-isopropylacrylamide) (AgNPs/PNIPAM) hydrogel was constructed by the in situ reduction of silver ions on a PNIPAM membrane, with polyvinylpyrrolidone (PVP) used as a surfactant [71]. The AgNPs were uniformly loaded on the hydrogel network, and the composite membrane was given good mechanical properties and reinforced swelling behavior. An enhanced SERS sensitivity together with an improved signal uniformity with a relative standard deviation (RSD) below 10% was achieved, and the pesticide detection concentration was pushed down to 10^−9^ M. Subsequently, the flexible AgNPs/PNIPAM hydrogel was applied to curved apple surfaces for the in situ qualitative and quantitative analysis of pesticide residues, with the analytical concentrations found to be lower than the European Union standards. This hydrogel’s swelling behavior facilitates analyte enrichment by absorbing target molecules into the hydrated network, bringing them closer to the embedded AgNPs and thus improving sensitivity on wet or irregular surfaces. This hydrogel strategy stands out for its straightforward fabrication and good mechanical compliance; however, its sensitivity remains moderate when compared with more elaborate architectures discussed below.

Subsequently, sandwich nanoarchitectures have been developed to boost performance through synergistic plasmonic coupling. A sandwich nanoarchitecture was built by the vacuum-filtration assembly of multiscale nanomaterials, in which Au nanoparticles were sandwiched between two Ti_3_C_2_T_x_ MXene layers supported on TEMPO-oxidized nanocellulose fibers (MX@Au@MX@TC film). Efficient charge transfer and numerous nanogaps were simultaneously provided by this layered structure, and vertical plasmonic coupling was enabled between Au nanoparticles and MXene nanosheets [72]. An ultra-low LOD of 10^−11^ M and an enhancement factor of 9.9 × 10^9^ were obtained for rhodamine 6 G, and an RSD of 2.29% was demonstrated. The substrate was further applied to the curved surface of apples, and thiram was directly detected at 1 ppm. The vertical plasmonic coupling generates dense, uniform hot spots, achieving low LOD and high signal uniformity for reliable detection of trace-level contaminants on curved food surfaces. Notably, this laminated design achieves an LOD two orders of magnitude lower than that of the hydrogel, along with far superior signal uniformity, although these gains come at the cost of a more complex multilayer fabrication process. In parallel, biomimetic replication offers an alternative route to enriching analytes through hierarchical surface textures. A rose-petal-like flexible substrate was fabricated by a two-step molding process, through which the unique three-dimensional micro/nanostructures of fresh rose petals were accurately replicated on polydimethylsiloxane [73]. Excellent hydrophobicity (141.3°) and a large specific surface area were endowed on the prepared substrate, and more hotspots were provided under a single laser shot. After the loading of silver nanoparticles, the flexible substrate was used for the direct sampling and detection of thiram and methyl parathion on apple peels, and the LODs were found to be 1.8 and 1.2 ng cm^−2^, respectively. The strong hydrophobicity from the hierarchical micro/nanostructures concentrates analyte droplets onto a small area, increasing local hotspot density for better sensitivity on dry or waxy food surfaces where aqueous droplets tend to spread. While the biomimetic approach provides facile access to hydrophobic, hotspot-rich structures conducive to direct sampling, its sensitivity is reported in areal mass units, which complicates direct comparison with solution-phase LODs.

More recently, a flexible Au@Prussian Blue (PB)@Ag nanoparticles–chitosan foam (Au@PB@Ag NPs-CFs) substrate with self-calibration functionality was designed and synthesized [74]. A PB layer was fully encapsulated between the Au core and the Ag shell, and a dual electromagnetic enhancement effect on both the internal-standard signal and the pesticide signal was realized. After signal calibration, the RSD was decreased from 30.34% to 11.24%. The water-dispersible Au@PB@Ag nanoparticles loaded on chitosan foam were used for the detection of thiram and thiabendazole, and LODs as low as 0.015 μM and 0.098 μM were reached, respectively. Owing to the flexibility and excellent uniformity of the substrate, the coffee ring effect was effectively addressed, and quantitative detection was enabled through direct swabbing, with spiked recovery rates ranging from 81% to 116.6%. By compensating for signal fluctuations, the embedded Prussian Blue internal standard makes this design ideal for swabbing-based on-site detection where consistent sample-substrate contact is difficult to guarantee. Here, the embedded PB internal standard markedly suppresses signal variation, illustrating a favorable trade-off: reproducibility is prioritized, whereas the absolute sensitivity remains intermediate between the hydrogel and the MXene-based laminate.

Moving beyond solution-processed nanomaterials, track-etched membranes have emerged as a versatile flexible platform for SERS substrate fabrication. Their well-defined cylindrical pores enable template-assisted synthesis of metal nanostructures with precisely controlled diameter, length, and density. For instance, silver-coated PET track-etched membranes were prepared via chemical immobilization of AgNPs on diethylenetriamine-modified surfaces, yielding a robust SERS platform [75]. Alternatively, gold nanostars were directly grown on ion-track-etched polycarbonate membranes through a one-step redox reaction, achieving an LOD of 10^−10^ M for R6G [76]. Furthermore, Ag-nanowire arrays electrodeposited within track-etched membrane pores spontaneously formed bundles upon drying, generating gap hot spots for enhanced SERS signals [77]. More recently, spike-like track-etched membranes coated with a continuous silver layer and functionalized with DNA aptamers demonstrated stable SERS performance in biological media [78]. This template strategy offers exceptional control over nanostructure geometry and straightforward scalability; its sensitivity is comparable to that of the hydrogel approach but still falls short of the MXene laminate, with the independent tunability of pore size and density constituting its principal advantage.

Moreover, mechanical robustness under bending is a routinely evaluated property of flexible SERS substrates. Most designs rely on hot spots formed between discrete nanostructures, which are susceptible to signal degradation upon bending due to gap widening. A recent study addressed this limitation by employing silver nanodendrites electrodeposited on a graphene-modified polyimide film [79], where hot spots are located at trunk-branch and branch-branch junctions within individual dendrites and remain unaffected by substrate deformation. The SERS signal remained stable across bending angles from 0° to 75°, enabling reliable detection of 10^−12^ M p-aminothiophenol and 6-thioguanine on curved surfaces. This bending-insensitive design offers a practical advantage for on-site detection on irregular food surfaces where mechanical deformation is inevitable.

Taken together, these representative studies reveal that no single material or structural design dominates across all performance metrics. The hydrogel approach excels in mechanical flexibility and simplicity, the MXene/nanocellulose laminate delivers the highest sensitivity and uniformity, the biomimetic replica facilitates direct analyte enrichment, the core–shell architecture with an internal standard enhances reproducibility, and track-etched membranes offer precise morphological control. The optimal choice therefore depends on the specific demands of the target application, such as LOD, signal uniformity, fabrication complexity, or compatibility with real food matrices. Through this comparative analysis, it becomes evident that the rational selection of plasmonic materials, two-dimensional nanomaterials, biomimetic architectures, and core–shell structures with embedded internal standards can address the key challenges faced by flexible SERS substrates in real-world food safety monitoring.

### 2.2. Engineering of Flexible Support Materials

The choice of flexible support material is critical to the performance of a SERS substrate. It affects the conformal contact with curved food surfaces, the ease of sampling, and the practicality of on-site detection. Various flexible supports have been investigated in recent years, including polymer films, natural biomaterials, and electrospun nanofiber membranes [80,81,82]. The following examples illustrate how different support architectures contribute to SERS performance. A carbon fiber cloth was chosen as a flexible and conductive support for the hydrothermal growth of cobalt oxide nanowires, onto which gold nanoparticles were photodecorated [83]. The resulting Au-NPs/Co_3_O_4_ nanowire carbon fiber cloth substrate was used to detect methylene blue on fish skin and fish muscle through a swab-sampling technique, achieving an LOD of 1.42 × 10^−10^ M. This design exploits the intrinsic conductivity of carbon fiber cloth to facilitate uniform nanowire growth, offering a robust scaffold for high-temperature synthesis. However, its relatively rigid woven structure may limit conformal contact on highly curved surfaces compared to softer polymeric alternatives. This rigidity trade-off means the carbon fiber cloth is best suited for flat or gently curved food surfaces such as fish fillets, rather than highly curved items like whole fruits.

In contrast to conductive fibrous supports, porous aerogels provide a distinct mechanism for signal enhancement through solvent-induced structural collapse. A self-crosslinked starch aerogel was prepared without any additional cross-linking agents, and silver nanoparticles were uniformly dispersed within the aerogel matrix [84]. The three-dimensional porous structure collapsed upon contact with water, generating dense hot spots and strong SERS enhancement. This aerogel was applied to detect thiabendazole on citrus surfaces by wipe sampling, with an LOD of 0.031 mg/L and recovery rates between 96.2% and 98.8%. Unlike the carbon fiber cloth, which relies on preformed nanowire arrays, the starch aerogel leverages a dynamic structural transition triggered by the sampling solvent itself, creating hot spots only at the moment of detection. This smart-responsive design yields good recovery rates, though the requirement for aqueous contact may limit its applicability to dry-surface sampling scenarios. The solvent-triggered collapse mechanism is particularly advantageous for moist or freshly washed food surfaces where residual water can be harnessed as part of the detection workflow, but less practical for dry-stored produce.

Moving to a more flexible and scalable platform, electrospun nanofiber membranes offer a different balance between ease of fabrication and detection performance. Cellulose acetate nanofiber membranes were fabricated by electrospinning and then coated with gold nanoparticles via magnetron sputtering [85]. The optimized AuNPs@cellulose acetate nanofiber substrate showed an LOD 10^−11^ M for 4-mercaptobenzoic acid (4-MBA), with an RSD of 9.5%. Thanks to the flexibility of the electrospun membrane, thiram residues on irregular apple surfaces were detected through an adhere-and-read method, reaching an LOD of 10^−7^ M. While electrospun nanofiber membranes offer good flexibility and scalability, alternative approaches inspired by natural structures have also attracted attention for their unique topographic features. A biomimetic flexible SERS substrate was produced by replicating the nanopore arrays of cicada wings onto a PDMS film, followed by silver nanoparticle deposition via magnetron sputtering [86]. This substrate exhibited an analytical enhancement factor of 4.2 × 10^5^ and a relative standard deviation of 7.3%. Combined with a portable Raman spectrometer, malachite green residues on apple and pear peel were detected under backside illumination, with an LOD of 10^−6^ M. The biomimetic PDMS substrate demonstrates good signal uniformity among the four designs, benefiting from the precisely replicated nanopore array. However, its enhancement factor is modest compared to typical metal-nanostructure-based substrates, and its real-sample LOD for malachite green is similar to that of the electrospun membrane for thiram, indicating that biomimetic replication alone may not yield the highest sensitivity without additional plasmonic optimization.

Taken together, these four support-material strategies each present distinct trade-offs. The carbon fiber cloth offers electrical conductivity and thermal stability suitable for hydrothermal synthesis, but its rigidity may limit conformal contact. The starch aerogel provides a responsive hot-spot generation mechanism with high recovery rates, yet its operation is inherently tied to aqueous conditions. The electrospun nanofiber membrane combines good flexibility, scalability, and high probe-molecule sensitivity, though real-sample performance still trails behind. The biomimetic PDMS replica achieves superior signal uniformity but with a lower enhancement factor. Across these comparisons, it is evident that no single support material universally outperforms others; the optimal choice depends on the specific demands of the target applications.

### 2.3. Performance Enhancement Strategies

Performance enhancement of flexible SERS substrates relies on various fabrication strategies beyond simple material selection [87,88,89]. These include template-assisted micropore structures, hydrogel shrinkage-mediated hotspot densification, electrochemical modulation [90], self-cleaning design [91], and acoustic streaming enrichment. Among physical structure engineering approaches, template-assisted micropore arrays and hydrogel shrinkage-mediated hotspot densification represent two distinct routes to enhancing SERS performance. For example, a flexible SERS patch was fabricated by replicating a PDMS microhole array through 3D-printed mold casting [92]. Into these microholes, an inverse opal photonic crystal (IOPC) hydrogel structure was constructed and subsequently modified with colloidal AgNPs. The periodic refractive index distribution of the 3D IOPC hydrogel generated high-density electromagnetic hot spots at the band edge, and an enhancement factor of 4.6 × 10^4^ was calculated. Due to the sticky nature of PDMS and the absorbent property of the hydrogel, pesticide molecules on irregular surfaces were efficiently extracted by a simple press and peeled-off procedure, with an extraction efficiency of 55.13 ± 8.85% on apple surfaces. The patch was applied to the detection of thiabendazole, triazophos, and chlorpyrifos on fruits and vegetables, and the LODs were determined to be 24.15, 37.56, and 4.21 ng cm^−2^, respectively. The combined adhesion and absorption mechanism makes this patch particularly effective for non-destructive sampling on delicate fruit skins where rigorous swabbing might cause damage.

**Figure 2:**
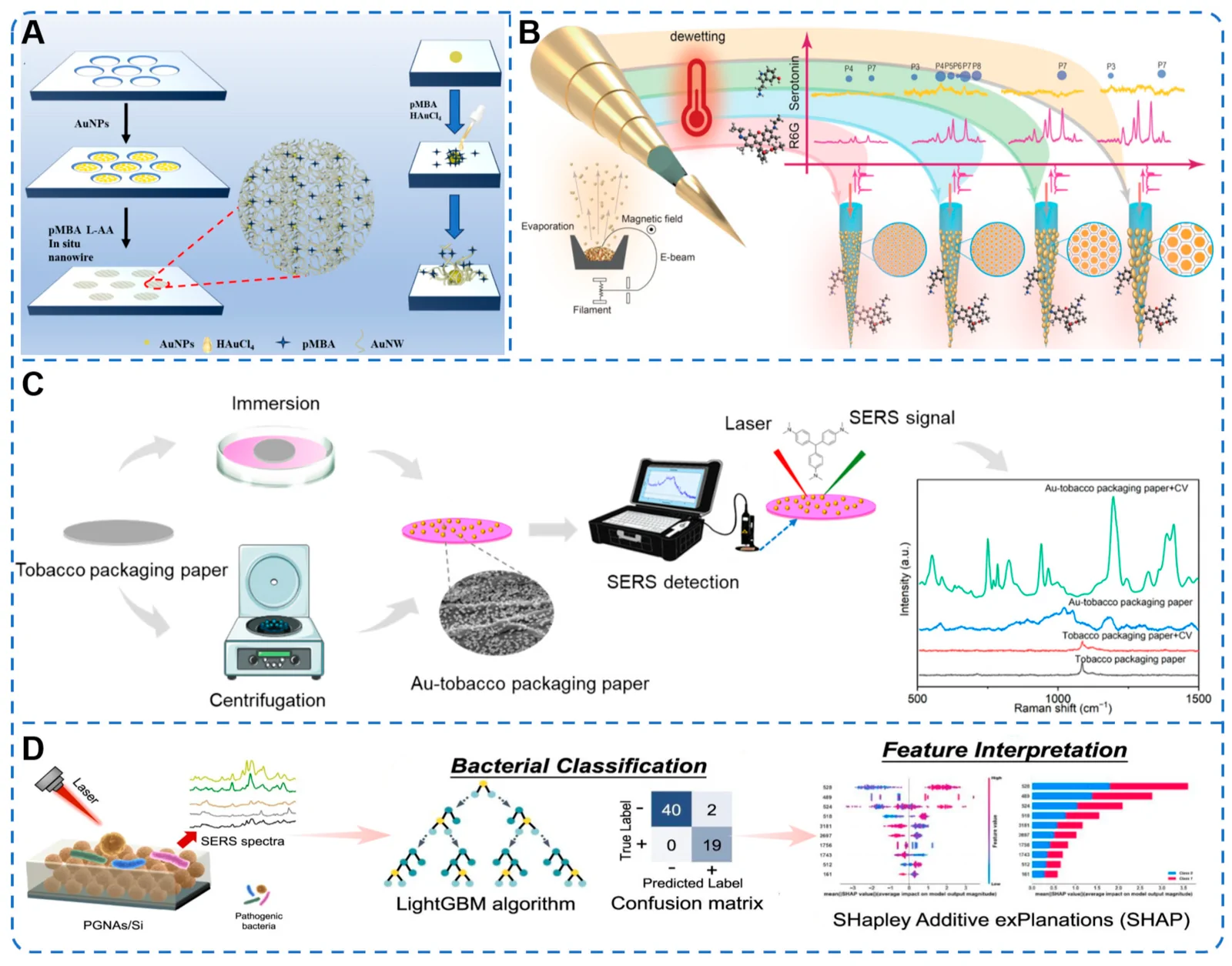
(**A**) Schematic illustration of the template-assisted fabrication of 3D AuNW networks. Reprinted with permission from Ref. [93]. Copyright 2025, American Chemical Society. (**B**) Schematic of tunable gold nanoislands on tapered fibers for through-fiber SERS detection. Reprinted with permission under a Creative Commons [CC BY-NC-ND 4.0] license from Ref. [104]. Copyright 2024, American Chemical Society. (**C**) Schematic illustration of the preparation of the Au-tobacco packaging paper substrate and the SERS detection process. Reprinted with permission under a Creative Commons [CC BY 4.0] license from Ref. [38]. Copyright 2022, MDPI. (**D**) Schematic of the dendrimer-based SERS platform and class-incremental learning for rapidly detecting pathogenic bacteria. Reprinted with permission under a Creative Commons [CC BY 4.0] license from Ref. [105]. Copyright 2024, Elsevier.

Moreover, Uniform nanoholes were fabricated in an aluminum film on a flexible PET film through the size-tunable organic nanodot array method [93]. Twenty-nanometer AuNPs were then incubated in these nanoholes for 12 h, grafting them onto the substrate as initial nucleation sites. Under the induction of 4-MBA, anisotropic in situ growth of gold nanowires was selectively triggered at the AuNP–substrate interface, forming a three-dimensional interconnected network (Figure 2A). The nanowire junctions generated intense plasmonic coupling, yielding numerous electromagnetic hot spots. When integrated into food packaging, the 3D gold nanowire platform enabled label-free detection of volatile biogenic amines such as cadaverine and putrescine in spoiled seafood, with LODs as low as 2.55 × 10^−14^ M and 2.09 × 10^−12^ M, respectively.

Additionally, an isotropic shrinkage strategy was introduced to a plasmonic nanoparticle-loaded hydrogel SERS sensor for robust and sensitive pesticide detection [94]. Ag@polyacrylamid hydrogels were prepared by chemical crosslinking and in situ silver deposition. Instead of air drying, the substrate was soaked in ethanol for 12 min to induce isotropic shrinkage. The shrinkage drew AgNPs closer together, intensifying hotspot distribution, and a 17-fold Raman signal increase for 4-nitrothiophenol was achieved. The LOD for 4-nitrothiophenol was calculated to be 2.21 × 10^−12^ M, and the RSD of the SERS signal was reduced to 8.04%, demonstrating improved homogeneity compared to air-dried substrates. The sensor was further applied to thiram and thiabendazole in liquid environments, with LODs of 5.26 × 10^−10^ g mL^−1^ and 3.00 × 10^−8^ g mL^−1^, respectively. On curved apple and grape surfaces, trace thiram and thiabendazole were also quantitatively detected, with recovery rates ranging from 82.50% to 115.35%. The isotropic shrinkage ensures uniform hotspot densification throughout the hydrogel volume, producing consistent signals even on curved surfaces where uneven pressure during sampling might otherwise cause signal variation. Comparing these two physical strategies, the IOPC hydrogel patch offers the advantage of efficient analyte extraction from irregular surfaces via a simple press-peel procedure, but its enhancement factor is modest. In contrast, the ethanol-induced shrinkage approach achieves a substantially lower solution-phase LOD and better signal uniformity, though it requires an additional solvent-soaking step that may complicate field deployment.

In addition to template and hydrogel shrinkage approaches, other strategies such as electrochemically assisted SERS (EC-SERS) [95], acoustic streaming, self-cleaning heterostructures [91], and biodegradable supports have also been explored to further broaden the applicability of flexible SERS substrates. For instance, a molecularly imprinted polymer-based EC SERS (MIP-EC-SERS) sensor was constructed on an AuNPs/indium tin oxide (ITO) electrode [96]. A polydopamine layer rich in imprinted cavities was fabricated on the AuNPs/ITO surface by electropolymerization, yielding a SERS substrate modified with MIP. Selective capture of acetamiprid by imprinted cavities provided the initial signal enhancement. Subsequently, applying a potential of +0.3 V promoted the interactions between acetamiprid and the SERS interface, further amplifying the SERS signals. The MIP-EC-SERS sensor achieved an LOD down to 3.2 nM, which was 13.6 times higher in sensitivity than detection without the applied potential. The practical applicability was confirmed by accurately testing acetamiprid in vegetable samples. Beyond electrochemical modulation, self-cleaning capability has emerged as another important strategy for improving the reusability and sustainability of flexible SERS substrates [97,98]. By incorporating photocatalytic semiconductors, target molecules adsorbed on the substrate can be degraded under UV irradiation, allowing the same substrate to be used for multiple detection cycles. A self-cleaning flexible SERS substrate was fabricated by electrospinning a TiO_2_-doped PVDF/PVP composite into a porous nanofiber felt, onto which Au@Ag core–shell nanorods were assembled via electrostatic layer-by-layer deposition [99]. The TiO_2_ nanoparticles embedded in the fiber framework provided photocatalytic degradation under UV light, while the porous structure created by PVP removal increased the surface area for analyte capture. The substrate was applied to detect methyl-parathion on apple surfaces through a pasting-extraction method, achieving an LOD of 0.037 ng/cm^2^. After five consecutive cycles of detection and UV photodegradation, the RSD of the characteristic peak intensity remained at 5.61%, demonstrating satisfactory reusability. Comparing these two dynamic strategies, the EC-SERS approach achieves higher sensitivity through active signal amplification, while the self-cleaning design prioritizes sustainability and reusability over raw sensitivity, with stable performance across five cycles. The self-cleaning capability is particularly valuable for field applications where replacing substrates between every measurement is impractical, enabling continuous monitoring with a single device.

Physical enrichment techniques have also been explored to improve detection sensitivity. A flexible SERS substrate based on surface acoustic wave-induced nanoparticle enrichment was proposed [100]. AuNPs in a 5 μL droplet were concentrated into compact nanoclusters on a PDMS film by acoustic streaming. The coffee-ring effect was effectively suppressed, and the nanoparticle distribution area was reduced by 90%. The substrate showed a minimum detectable concentration of 10^−13^ M for rhodamine 6G and an RSD of 3.59% for crystal violet detection. The flexible PDMS film was applied to detect thiram residues on apple surfaces, achieving an LOD of 10^−8^ M. Although the acoustic streaming method achieves good signal uniformity and a low probe-molecule LOD, its real-sample LOD for thiram is several orders of magnitude higher, suggesting that analyte extraction efficiency from food surfaces remains a limiting factor. Despite this limitation, the acoustic approach offers a contactless enrichment mechanism that avoids direct substrate contamination, which is advantageous for serial sampling of multiple food items without cross-contamination.

Several integrated platforms have been developed that combine novel substrate architectures with advanced data analysis methods to address specific detection challenges [101,102,103]. A tapered optical fiber decorated with tunable gold nanoislands was fabricated by solid-state dewetting, enabling remote SERS detection through the fiber itself (Figure 2B) [104]. The nanoisland patterns with controlled size and density were systematically studied, and serotonin was detected with an LOD of 10^−7^ M in a through-fiber configuration, representing the lowest value reported for such a scheme. In a different approach, a low-cost paper-based SERS substrate was prepared by adsorbing AuNPs onto commercial tobacco packaging paper (Figure 2C) [38]. The flexible Au- tobacco packaging paper substrate was used to wipe pork surfaces spiked with *Staphylococcus aureus* (*S*. *aureus*) and *Shigella flexneri*, and characteristic Raman fingerprints of both pathogens were obtained using a portable spectrometer. Principal component analysis (PCA) achieved 100% classification accuracy, demonstrating the feasibility of rapid on-site bacterial screening. Furthermore, a dendrimer-based platform was constructed by assembling AuNPs with poly(amidoamine) on a silicon wafer, forming nanoassemblies with ultra-small internal nanogaps (Figure 2D) [105]. Four pathogenic bacteria were detected at concentrations as low as 10 CFU/mL in water, milk, and dietary supplement samples. A class-incremental learning model using the LightGBM algorithm was employed to classify the SERS spectra, achieving an accuracy above 93% and enabling rapid discrimination of multiple pathogens in complex matrices. Although this platform employs a rigid silicon wafer as the supporting substrate rather than a flexible material, its key contribution lies in demonstrating the feasibility of combining ultra-small nanogap engineering with machine learning-based spectral classification for reliable pathogen identification in complex food matrices. Among these three integrated platforms, the dendrimer-based approach achieves high sensitivity and incorporates machine learning for automated pathogen identification, whereas the optical fiber design enables remote detection but with a higher LOD, and the paper-based substrate offers the lowest cost and simplest operation at the expense of sensitivity. The paper-based substrate’s simplicity and low cost make it the most practical option for routine screening in resource-limited settings, despite its comparatively modest sensitivity.

These diverse strategies reveal a clear trade-off between sensitivity, operational simplicity, and reusability. Physical structure engineering, particularly the nanowire junction approach, achieves the lowest LODs but often requires multi-step fabrication. Dynamic strategies such as EC-SERS and acoustic streaming provide real-time control and good uniformity but add instrumental complexity. Self-cleaning designs prioritize long-term reusability, while integrated platforms with machine learning offer automated pathogen classification at the cost of higher computational demand. These diverse strategies collectively advance the development of high-performance, field-deployable flexible SERS platforms for food safety monitoring.

It is worth noting that the specificity of flexible SERS sensors deserves equal attention alongside sensitivity. Compared with conventional rigid SERS substrates, flexible SERS sensors do not inherently possess higher chemical specificity, as both rely primarily on the molecular fingerprint provided by Raman scattering [106]. However, the unique sampling capabilities of flexible substrates can indirectly improve apparent specificity in practice. For instance, the conformal contact and wiping action of flexible substrates physically enrich target analytes from irregular surfaces while excluding bulk matrix components, thereby reducing background interference. Moreover, when flexible substrates are integrated with specific recognition elements such as MIP, aptamers, or antibodies, their specificity can rival that of established analytical techniques. The MIP-EC-SERS sensor discussed above exemplifies this synergy, where imprinted cavities selectively capture acetamiprid, and the applied potential further amplifies the signal [96]. Therefore, the specificity of flexible SERS sensors is considered sufficient for practical food safety monitoring when appropriate recognition strategies are incorporated.

## 3. Applications of Flexible SERS Substrates in Food Safety Monitoring

Flexible SERS substrates have been widely applied to food safety monitoring, leveraging their ability to conform to irregular surfaces and enable direct, on-site detection (Table 1) [107,108,109]. In the context of food safety, where complex matrices containing pigments, lipids, proteins, and other interfering species are common, achieving high specificity is challenging for all SERS-based approaches. Flexible SERS sensors, when relying solely on bare plasmonic nanostructures, exhibit specificity comparable to that of conventional rigid SERS substrates. However, the flexibility-enabled sampling strategies (swabbing, pressing, wrapping) provide a unique advantage: they allow physical separation of the target from bulky contaminants during the sampling step, thereby improving the signal-to-noise ratio and apparent specificity. Furthermore, as highlighted in Section 2.3, the integration of molecular recognition elements or advanced data analytics can elevate specificity to levels suitable for regulatory compliance. This section organizes the applications by sampling and detection modes rather than by target analyte, highlighting the versatility of flexible SERS platforms. Four major modes are discussed: swabbing-based detection on irregular surfaces, in situ detection, enrichment-assisted detection for trace analytes, and multiplexed analysis.

### 3.1. Swabbing-Based Detection on Irregular Surfaces

Flexible SERS substrates enable direct swabbing of irregular food surfaces through conformal contact, where target analytes are physically transferred onto the sensing area without sample pretreatment [130,131,132]. This approach has been successfully applied to a wide range of food matrices, including fish skin and fillets, fruit and vegetable peels, and poultry products. The following representative studies illustrate how swabbing-based detection has been implemented across different hazard categories using various flexible substrate designs.

**Figure 3:**
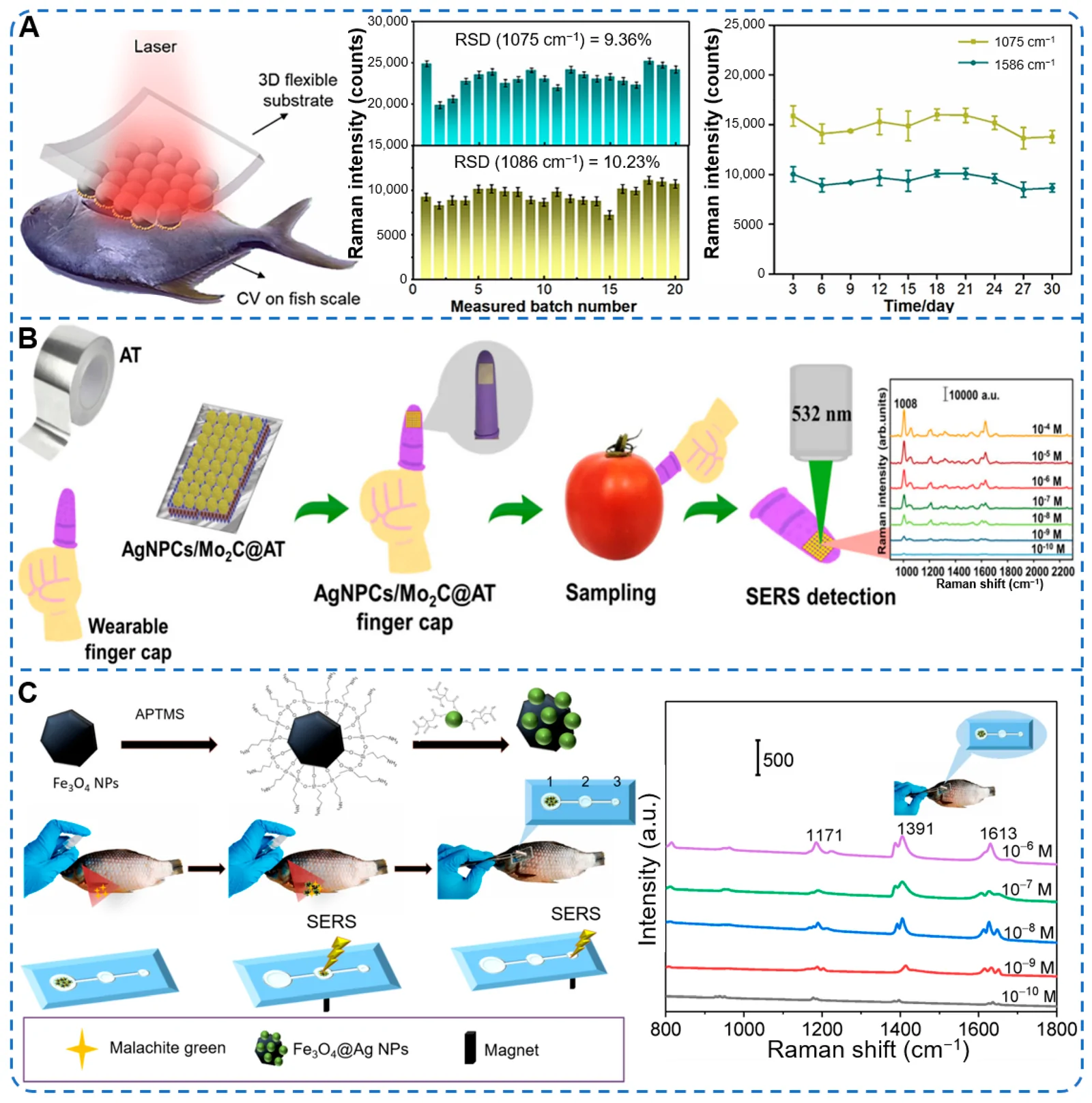
(**A**) Schematic of SERS detection of crystal violet on fish skin using a 3D flexible PDMS substrate. Reprinted with permission from Ref. [110]. Copyright 2024, American Chemical Society. (**B**) Illustration of the wearable AgNPCs/Mo_2_C@adhesive aluminum tape finger cap for SERS sampling and detection. Reprinted with permission from Ref. [113]. Copyright 2026, American Chemical Society. (**C**) Schematic of the magnetic–plasmonic composite and its application for sensing malachite green from fish skin by magnetic fluid SERS, and SERS spectra of malachite green. Reprinted with permission under a Creative Commons [CC BY 4.0] license from Ref. [116]. Copyright 2022, MDPI.

A three-dimensional flexible SERS substrate was fabricated by a layer-by-layer transfer strategy, through which gold nanospheres were assembled onto a polystyrene (PS) microsphere array on PDMS [110]. The Au nanospheres/PS/PDMS substrate was placed in direct contact with the surface of fish spiked with crystal violet, and the laser was focused through the transparent PDMS to acquire SERS spectra (Figure 3A). An LOD of 0.028 ppm was achieved on fish surfaces, and a linear response was obtained in the concentration range from 0.05 to 10 ppm, with a fitting coefficient of 0.97.

In another study, a different supporting material was explored to achieve both flexibility and reusability [111]. AgNPs were deposited on polyamide fabric via magnetron sputtering. The resulting Ag@polyamide substrate was moistened with water and wiped over the contaminated surface of a Pacific saury to which malachite green had been applied. An LOD as low as 7.8 × 10^−8^ M was reached, and a linear relationship between SERS intensity and the logarithm of malachite green concentration was observed over the range from 10^−4^ to 10^−7^ M, with a coefficient of determination of 0.86. The substrate also showed an RSD of 5.4% across ten random positions and maintained its performance after five cycles of detection and cleaning. The reusability of the fabric-based substrate reduces per-test cost and waste generation, which is a practical advantage for routine screening of multiple fish samples in processing facilities. Beyond the choice of support material, the surface morphology of the plasmonic nanostructures themselves was also engineered to enhance hotspot density. Aqua-regia-treated silver nanowires were embedded into a sandpaper-templated PDMS matrix to form a flexible patterned SERS substrate [112]. The chemical etching increased the surface roughness of the nanowires, generating a higher density of SERS hotspots, while PDMS encapsulation prevented nanowire oxidation. The substrate was pressed onto and peeled from fish skin to collect residues of crystal violet and malachite green. LODs of 89 nM for crystal violet and 54 nM for malachite green were achieved, with analytical enhancement factors of 2.1 × 10^7^ and 6.21 × 10^6^, respectively. Over a 60-day storage period, the substrate retained 89.22% of its original signal intensity, with an RSD of 3.68%.

The swabbing approach has also been widely adopted for pesticide residue detection on fruits and vegetables. In these applications, flexible SERS substrates are pressed against or wiped over curved surfaces to collect target molecules directly. A wearable SERS-active finger cap was fabricated by decorating silver nanopopcorns (AgNPCs) and molybdenum carbide (Mo_2_C) nanoparticles on adhesive aluminum tape [113]. The hybrid AgNPCs/Mo_2_C@adhesive aluminum tape substrate was worn on a finger and pressed onto tomato and chili surfaces using a simple paste-press-peel procedure (Figure 3B). Fipronil residues were efficiently collected from both exocarp and mesocarp regions. An LOD of 1.16 × 10^−10^ M was achieved on glass slides, and values of 1.65 × 10^−10^ M and 1.72 × 10^−10^ M were obtained on tomato and chili exocarps, respectively. The substrate also showed good signal uniformity (RSD = 8.81%) and retained 70% of its original activity after 14 days. Moreover, a paper-based SERS substrate was prepared by in situ growth of AgNPs on filter paper via a silver mirror reaction [114]. Two sampling modes were employed: capillary-assisted SERS and direct wiping. For the wiping method, the flexible Ag@filter paper substrate was moistened with methanol and wiped over the apple surface to collect thiram residues. An LOD of 0.005 ng/cm^2^ was achieved with capillary-assisted-SERS, while the wiping method provided faster sampling (under 1 min) with a higher LOD. The substrate was prepared in 7 min and remained stable at room temperature for 40 days.

The swabbing strategy has also been applied to antibiotic residues on poultry products. A flexible 3D plasmonic nano-cauliflower (PNC) substrate was fabricated by drop-casting gold nanostars onto commercial adhesive tape [115]. The hydrophobic tape surface concentrated the hydrophilic gold nanostars into a 3D architecture upon drying, generating abundant hotspots. The PNC substrate was pressed onto chicken wing skin spiked with ciprofloxacin and ampicillin, left in contact for 5 min, and then peeled off for SERS measurement. LODs of 3.3 nM for ciprofloxacin and 1.5 nM for ampicillin were achieved. Simultaneous detection of both antibiotics in a mixture was also demonstrated on chicken skin, with clear spectral discrimination even at 50 nM. The substrate exhibited an RSD below 5% and remained stable for over six months.

Furthermore, a magnetic-fluidic SERS device was developed by decorating AgNPs onto Fe_3_O_4_ NPs to form magnetic-plasmonic AgMNPs [116]. The AgMNPs suspension was sprayed directly onto fish skin contaminated with malachite green, and then collected onto a microfluidic chip with three cascaded reservoirs under an external magnetic field (Figure 3C). The confinement of AgMNPs into progressively smaller reservoirs concentrated the target analytes and enhanced the SERS signal. An LOD down to 10^−12^ M was achieved for malachite green on fish surfaces, demonstrating the potential of magnetic fluid-based swabbing for on-site aquaculture safety monitoring. The magnetic confinement concentrates both the plasmonic nanoparticles and captured analytes into a small detection volume, achieving ultrahigh sensitivity without requiring precise alignment of a laser focal spot, which is a key advantage for portable Raman systems with fixed optics.

### 3.2. In Situ Detection

In situ SERS detection enables direct analysis of contaminants on food surfaces without sample collection or laboratory pretreatment. This approach relies on the conformal contact between a flexible substrate and the target surface, allowing spectra to be acquired directly from the sample itself [133]. SERS detection has been performed directly on target substrates without sample pretreatment. Inverted pyramidal structures were imprinted on a PET film by UV nanoimprinting, and AgNPs were deposited onto a thin aluminum interlayer to form an AgNPs/Al/pyramid PET substrate [117]. The substrate was attached directly onto crucian carp skin to which 10^−6^ M methylene blue had been applied, and SERS spectra were acquired in transmission mode (back-side illumination). This configuration achieved an enhancement factor of 9.3 × 10^11^ in transmission mode, and methylene blue residues on fish surfaces were reliably detected, validating the applicability of transmissive flexible substrates for on-site food safety monitoring. The back-side illumination configuration allows the substrate to remain in contact with the food surface during measurement, eliminating the need to transfer analytes to a separate detection zone and streamlining the on-site workflow.

**Figure 4:**
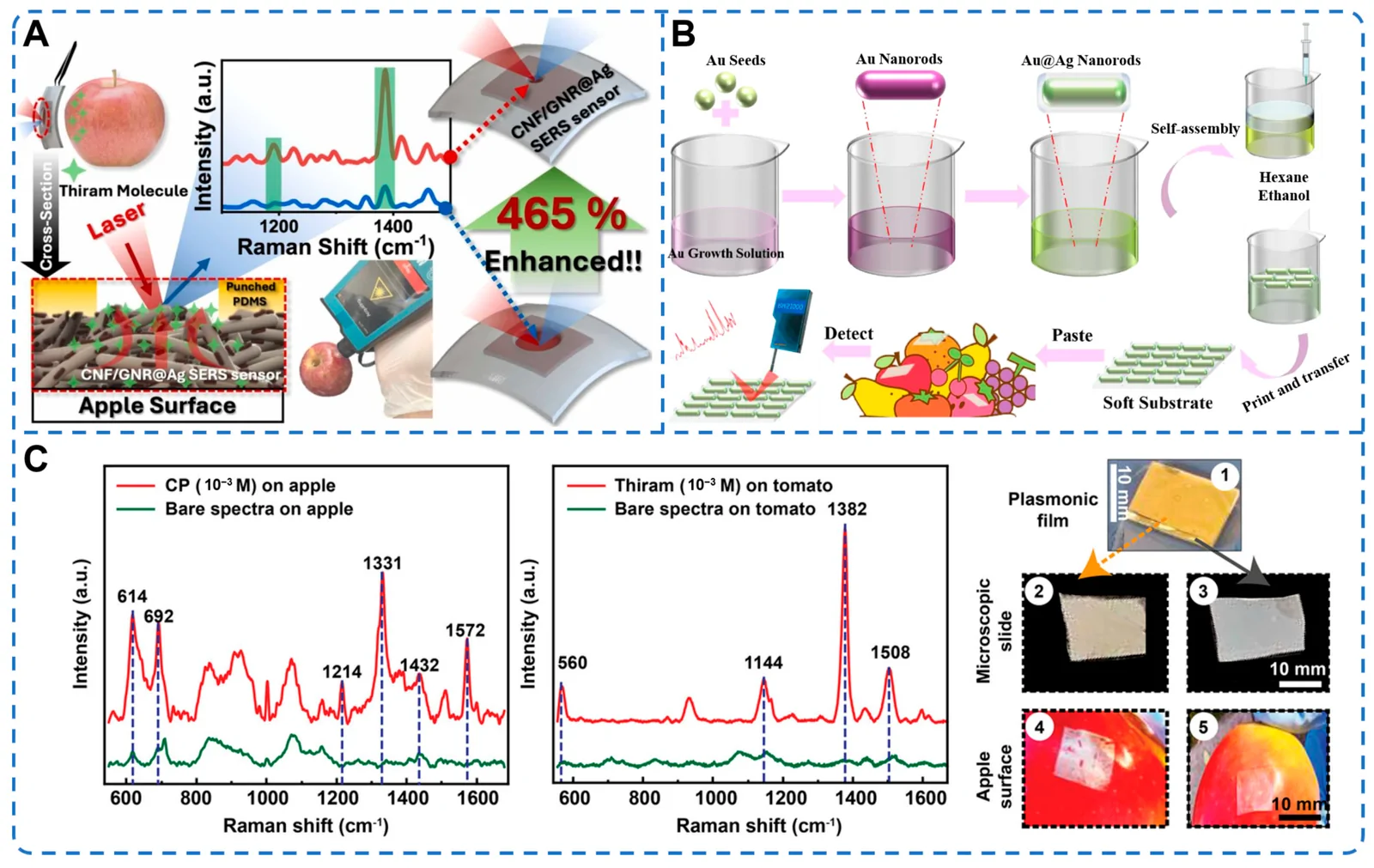
(**A**) Schematic of sensitive, on-site pesticide detection using a flexible CNF/GNR@Ag SERS sensor. Reprinted with permission under a Creative Commons [CC BY-NC] license from Ref. [118]. Copyright 2025, Elsevier. (**B**) Illustration of the fabrication of the Au@AgNRs array substrate and its application in pesticide residue detection on fruits/vegetables. Reprinted with permission under a Creative Commons [CC BY 4.0] license from Ref. [120]. Copyright 2022, MDPI. (**C**) Detection of chlorpyrifos on the surface of apple, detection of Thiram on the surface of tomato, and the schematic illustration of the universal transfer process of the plasmonic film. Reprinted with permission under a Creative Commons [CC BY 4.0] license from Ref. [121]. Copyright 2024, Wiley-VCH.

In a related approach, molecule enrichment was integrated into the in situ detection procedure to further improve sensitivity. Cellulose nanofibers (CNFs) were mixed with gold nanorod (GNR)@Ag core–shell structures and deposited by vacuum filtration to form a flexible CNF/GNR@Ag membrane [118]. A hole-punched PDMS layer was aligned onto the membrane, so that evaporation occurred exclusively within the confined hole area, driving capillary flow that concentrated target molecules. The sensor was pre-wetted with ethanol and attached directly to apple and chili pepper surfaces sprayed with thiram. Using a portable Raman spectrometer, thiram was detected at concentrations as low as 10^−11^ M on apple surfaces, and the localized evaporation enrichment improved SERS intensity by up to 465% compared with a 3 mm evaporation diameter (Figure 4A). Another design strategy employed a biopolymer-based composite gel to achieve label-free, nondestructive in situ detection [119]. Silver nanocubes (AgNCs) were hydrothermally synthesized and blended with a sodium alginate–chitosan (SA-CTS) composite gel, followed by freeze-drying to form a porous SA-CTS@AgNCs flexible substrate. The gel was placed directly onto apple peels where thiram residues had been spiked, and SERS spectra were collected using a handheld Raman spectrometer at 785 nm. An LOD of 0.055 mg/L was achieved for thiram on apple surfaces, with point-to-point RSD of 4.2% and batch-to-batch RSD of 6.8%. After 45 days of storage, the substrate retained 84.40% of its original SERS activity, demonstrating long-term stability for field deployment. The biopolymer gel matrix provides a biodegradable and skin-safe alternative to synthetic adhesives, which is advantageous for direct-contact food inspection where material safety is a regulatory concern.

In parallel with substrate designs based on adhesive tapes and hydrogels, transparent flexible SERS substrates have also been developed to enable back-side illumination during in situ detection [120]. Flexible and transparent SERS substrates were fabricated by transferring Au@Ag core–shell nanorod arrays onto silicone membranes, where the Au@AgNRs arrays were prepared by a liquid–liquid interface self-assembly method (Figure 4B). The prepared substrate could be readily pasted onto the surface of strawberries, apples, and mushrooms. Using a portable Raman spectrometer with 785 nm excitation from the back side, thiram residues were directly detected on these irregular surfaces, and a limit of detection as low as 2 ng/cm^2^ was reached, which is far below the maximum residue limit stipulated by regulatory agencies. The transparency of the silicone membrane enables visual alignment of the substrate over the target area, facilitating accurate positioning on irregular food surfaces during handheld operation.

Distinct from transparent silicone-based substrates, natural biopolymer supports have been explored to improve both biocompatibility and hotspot uniformity [134,135]. A flexible SERS substrate was fabricated by incorporating Ag@Au core–shell nanoparticles into a bacterial nanocellulose matrix through vacuum filtration [136]. The ultrathin Au shell (2 nm) protected the Ag core from oxidation, while the 3D nanofiber network of bacterial nanocellulose secured uniform loading of nanoparticles and promoted adsorption of target molecules. The substrate was pressed onto apple and pear peels after ethanol treatment, and thiram residues were detected in situ with LODs of 0.0498 ppm and 0.0621 ppm, respectively. The substrate also maintained stable SERS intensity after 57 days of ambient storage.

Furthermore, hydrogel-based flexible substrates have been designed to combine intimate surface conformity with effective analyte enrichment [137,138,139]. A series of chitosan hydrogel-based SERS substrates was synthesized by in situ growth of silver nanoparticles AgNPs onto glutaraldehyde-crosslinked chitosan hydrogels, where the abundant hydroxyl and amino groups enabled electrostatic adsorption of silver ions and controlled nanoparticle growth [140]. The hydrophilic hydrogel was wetted with ethanol and adhered to contaminated fruit surfaces—including cherry, tomato, plum, and banana—for 5 min via a simple stick-and-read method. An LOD of 10^−7^ M was consistently achieved across all tested fruits, and the optimized substrate exhibited an enhancement factor of 1.2 × 10^7^ and an RSD of 8.14%, while retaining satisfactory performance after 200 bending cycles. Alternatively, a flexible plasmonic film with engineered micro-well arrays was fabricated by growing dendritic silver nanostructures on PDMS [121]. The film was optically transparent and conformally attached to fruit surfaces (Figure 4C). Using a handheld Raman spectrometer, thiram residues were detected directly on apples with a limit of detection of 10^−6^ M, while confocal Raman achieved an LOD as low as 10^−13^ M. The micro-well structure also enabled analyte enrichment via the pinning effect during droplet evaporation, contributing to the ultrahigh sensitivity.

Beyond substrate-level designs, recent efforts have advanced toward integrating flexible SERS sensors into functional food packaging systems for in situ monitoring. A nanostructured SERS sensor integrated into a stretchable and antimicrobial wrapper was recently developed [141]. The sensor was fabricated by transferring a Au nanostructure array onto electrospun thermoplastic polyurethane fibers by drop-casting of Ag nanoparticles to generate dense SERS hotspots. The wrapper, infused with curcumin, exhibited antimicrobial activity of 99.99% against *S. aureus* and 99.9% against *E. coli*. It was directly applied onto the surfaces of salmon, beef, pork, and oranges for non-destructive in situ detection of purines, proteins, lipids, β-carotene, and thiram. Furthermore, temporal SERS monitoring of dimethyl disulfide peaks enabled real-time tracking of spoilage progression, while the antimicrobial wrapper effectively delayed microbial growth and preserved food quality over 15 days. This work exemplifies the transition of flexible SERS from a standalone sensing substrate toward an integrated smart packaging platform capable of simultaneous monitoring and preservation, representing a promising direction for practical on-site food safety applications.

### 3.3. Enrichment-Assisted Detection for Trace Analytes

Enrichment-assisted detection has been recognized as an effective route to improve the sensitivity of flexible SERS substrates for trace analytes in complex food matrices [142,143]. The weak affinity between target molecules and SERS surfaces often limits direct detection at low concentrations, especially in liquid-phase samples containing interfering substances. Various strategies have been developed to address this limitation by actively concentrating analytes onto SERS hotspots prior to or during spectral acquisition [144]. These include electrical assistance through electro-driven adsorption or dielectrophoretic manipulation, physical enrichment via hydrogel swelling and molecular sieving, optical fiber probes for direct immersion in liquid matrices, and catalytic signal amplification using nanozymes. Representative examples covering pesticide residues, veterinary drugs, and foodborne pathogens are discussed below.

A magnetic Fe_3_O_4_@ZIF-8@Ag substrate was fabricated, in which Ag nanoparticles were grown on the surface of a ZIF-8 shell coating a Fe_3_O_4_ core [122]. The substrate was used as both an adsorbent and a SERS platform, and an external magnetic field was applied for rapid separation. In the electro-driven adsorption SERS experiment, a potential of −0.5 V was applied to the droplet containing acetamiprid, and effective adsorption was achieved within 7 min. The electro-driven SERS offered 9 times higher sensitivity compared with normal SERS, with an LOD down to 4 nM. The reliability of this strategy was confirmed in peach and cowpea samples. The combination of magnetic separation and electro-driven adsorption enables rapid analyte enrichment within minutes without complex fluidic systems, making it suitable for on-site sample pretreatment.

In a similar electrical-assistance context, dielectrophoresis was adopted to concentrate bacterial cells directly onto a flexible SERS-active electrode. A PET/ITO foil was modified by dielectric barrier discharge and then coated with a 70 nm silver layer via physical vapor deposition, forming a PET/ITO/Ag flexible platform [123]. An alternating electric field was applied between the flexible platform and a central metal wire, generating a negative dielectrophoretic force that directed *Escherichia coli* (*E. coli*) cells toward the SERS-active surface within 3 min. Quick isolation, concentration, and label-free identification of bacteria were thus realized on a single device. This system was further demonstrated for rapid, cultivation-free detection of *E. coli* in urine and apple juice samples, providing new opportunities for pathogen screening.

In addition, hydrogel-based enrichment strategies have also been developed to achieve sensitive SERS detection through physical adsorption and molecular sieving effects. A flexible SERS substrate was fabricated by embedding AuNPs into a polyethylene glycol diacrylate (PEGDA) hydrogel matrix through UV polymerization [124]. Dimethoate molecules were premixed with the AuNP-hydrogel precursor solution before polymerization, ensuring uniform distribution of the target analyte within the hydrogel network. The hydrogel was then applied directly onto olive surfaces, where dimethoate residues were absorbed and concentrated through hydrogel swelling. After drying, the shrinkage of the hydrogel brought AuNPs into closer proximity, generating dense hotspots. An LOD of 3.01 ppb was achieved using a laboratory spectrometer, and a comparable LOD of 3.13 ppb was obtained with a portable Raman spectrometer. The premixing of target molecules within the hydrogel precursor ensures homogeneous analyte distribution, while the subsequent drying-induced shrinkage generates hotspots without external equipment, enabling consistent performance on portable instruments. In another hydrogel-based design, a flexible SERS chip was constructed by incorporating silver nanoflowers into a polyvinyl alcohol (PVA) hydrogel through freeze–thaw cycling [125]. The porous hydrogel network acted as a molecular sieve, excluding large macromolecules such as proteins and lipids while allowing small-molecule veterinary drugs to diffuse into the interior. The chip was immersed in milk samples spiked with enrofloxacin at 30 °C for 20 min, during which enrofloxacin molecules were enriched within the hydrogel matrix through passive diffusion. After drying, the shrinkage of the hydrogel intensified the electromagnetic hotspots between adjacent silver nanoflowers. An LOD of 47.96 ppb was achieved for enrofloxacin in milk, with recovery rates ranging from 88.3% to 114% and RSDs between 1.78% and 7.83%.

In contrast to the passive diffusion mechanism of hydrogels, alternative strategies have integrated optical fiber probes or catalytic signal amplification to achieve enrichment and signal enhancement in complex liquid matrices. An optical fiber SERS probe was prepared by a laser-induced evaporation self-assembly method, through which GNR clusters were patterned on the fiber facet [145]. The fiber probe was simply immersed in tea soup contaminated with thiram and paraquat, and SERS spectra were acquired directly without any sample pretreatment. Numerous hotspots provided by the GNR clusters on the fiber facet enabled LODs as low as 1.0 μg/kg for thiram and 10.0 μg/kg for paraquat, with RSDs below 10%. Recovery rates between 85.0% and 110.0% were obtained, demonstrating the accuracy of this in situ detection method in complex liquid-phase systems. In a different strategy, enzymatic signal amplification was employed to improve the sensitivity of pathogen detection. A bifunctional Au@Pt core–shell nanozyme was synthesized, where the ultrathin Pt shell catalyzed the oxidation of 3,3′,5,5′-tetramethylbenzidine (TMB) into Raman-active oxTMB, and the Au core served as the SERS substrate to enhance the oxTMB signal [146]. The Au@Pt nanozyme was conjugated with anti-*Salmonella* antibodies and combined with immunomagnetic beads to form sandwich immune complexes. After magnetic separation, the complexes catalyzed TMB oxidation, and the SERS signal of oxTMB was recorded. An LOD of 10 CFU mL^−1^ was achieved for *Salmonella typhimurium* in milk samples using a portable Raman spectrometer.

### 3.4. Multiplexed Analysis

Multiplexed analysis has emerged as a key direction for flexible SERS substrates, enabling simultaneous detection of multiple hazards or intelligent resolution of complex spectral data through encoding strategies, machine learning, or matrix-effect correction [147,148]. Within the scope of this review, multiplexed analysis encompasses two distinct analytical capabilities: multi-target detection, where a SERS platform is applied to detect or identify multiple analytes either simultaneously through distinct spectroscopic signatures or sequentially across separate measurements; and multi-class spectral classification, where machine learning or chemometric algorithms discriminate between different analytes, bacterial species, or sample categories based on spectral pattern recognition. These two capabilities are not mutually exclusive. Some platforms integrate both, as exemplified by a microwell plate-integrated glass-silver nanoforest SERS platform that primarily demonstrates multi-class spectral classification via PCA of *E*. *coli*, *Salmonella*, and *S*. *aureus*, while also showing the ability to resolve species-specific peaks in mixed samples, which aligns with multi-target detection [149].

Multi-target detection relies on the intrinsic ability of SERS to resolve distinct vibrational fingerprints of different analytes, whether measured concurrently or individually. A durian-shaped multilayer core–shell Fe_3_O_4_@Au@Ag@Au composite was synthesized as a SERS substrate for flow magnetic detection of pesticide residues [150]. The Fe_3_O_4_ core provided superparamagnetism, while the outermost Au thorn layer generated abundant hot spots, yielding an enhancement factor of 3.01 × 10^7^. A flow magnetic detection method was proposed, in which the substrate suspension was dropped onto contaminated food surfaces and recollected by an external magnet within 5 s. LODs of 0.13 and 0.18 ng/cm^2^ were achieved for malachite green on fish surfaces and thiram on apple surfaces, respectively. Moreover, a regenerable AgNPs-CdS nanowires/nanofilm SERS substrate was constructed by interfacial confined self-assembly for multiplexed detection of carbamate pesticides [151]. CdS nanowires were in situ grown on the nanofilm to provide loading sites, and AgNPs were deposited as hot spots. The substrate was applied to simultaneously detect metolcarb, carbaryl, and aldicarb-sulfone in cherry tomatoes, pears, and apples. LODs of 0.00081, 0.00081, and 0.00072 mg/L were obtained for the three analytes, respectively, with coefficients of determination above 0.991. The substrate was reused for three cycles, demonstrating satisfactory regenerability. The simultaneous detection of three carbamate pesticides with regeneration capability makes this platform suitable for cost-effective routine monitoring of multiple pesticide residues on fresh produce. Both the durian-shaped Fe_3_O_4_@Au@Ag@Au composite and the AgNPs-CdS nanowires substrate exemplify multi-target detection, where multiple analytes are identified concurrently via their distinguishable SERS fingerprints in a single measurement.

**Figure 5:**
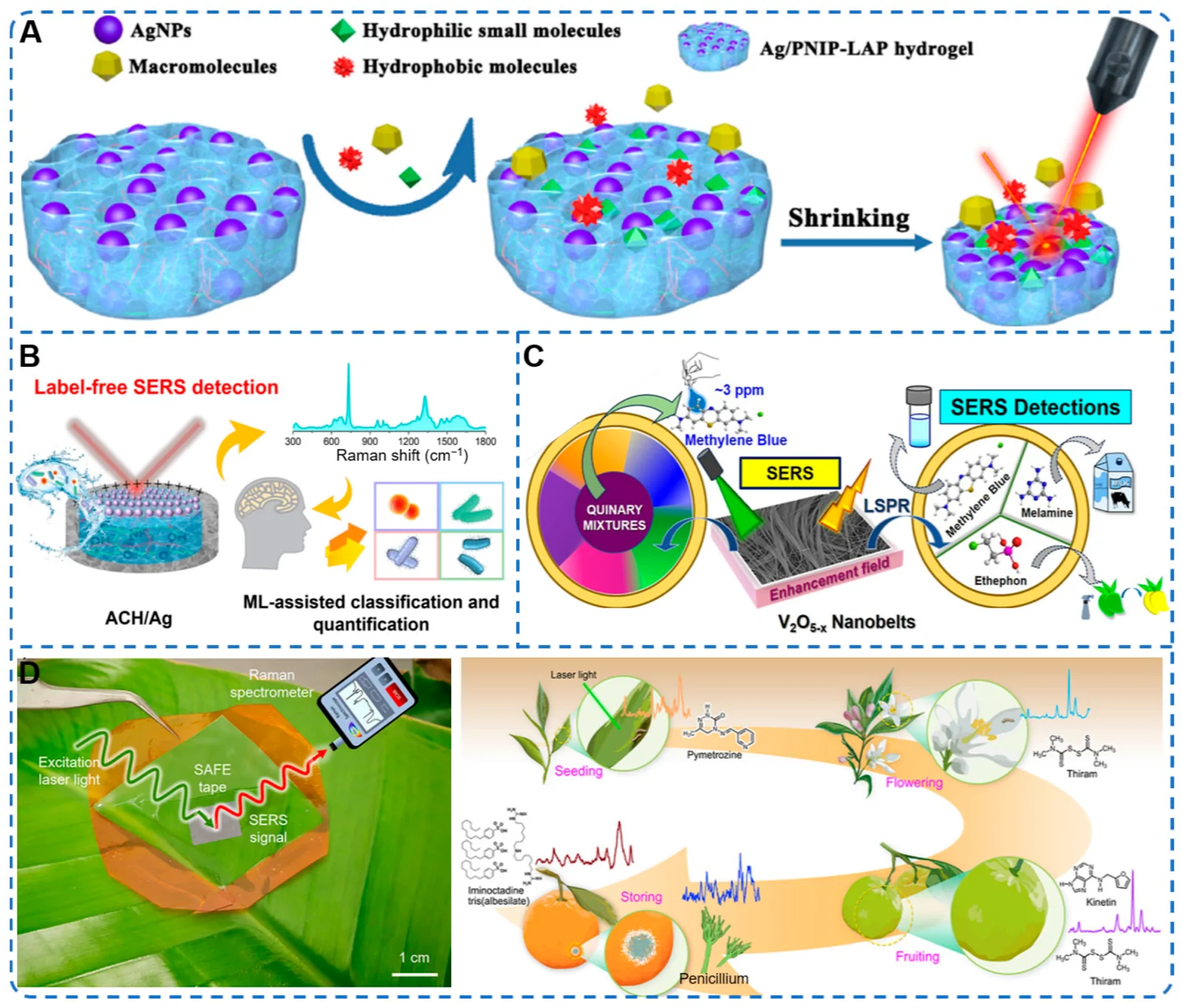
(**A**) Schematic illustration of the separation and SERS detection mechanism for hydrophilic small molecules within the Ag/PNIP-LAP hydrogel network. Reprinted with permission from Ref. [126]. Copyright 2023, American Chemical Society. (**B**) Construction of a flexible porous agarose/chitosan/Ag SERS platform and its application in machine-learning-assisted classification of multiple pathogenic bacteria. Reprinted with permission from Ref. [128]. Copyright 2026, American Chemical Society. (**C**) Illustration of Utilization of flexible V_2_O_5-x_ nanobelts as SERS tweezers for the highly sensitive and selective detection of quinary mixtures. Reprinted with permission from Ref. [153]. Copyright 2025, American Chemical Society. (**D**) Schematic diagram of the SAFE tape design, fabrication, and its function for in vivo SERS-active functional elastomeric monitoring of agrochemicals throughout plant growth stages. Reprinted with permission from Ref. [129]. Copyright 2026, American Chemical Society.

Substrate engineering and recognition chemistry offer alternative routes to multi-analyte SERS analysis in complex food matrices. A SERS-activated molecularly imprinted capillary sensor was constructed by modifying AgNPs onto the inner wall of glass capillaries, which served as the identification unit [152]. A dual-template imprinted polymer was synthesized through bulk polymerization and surface imprinting, with ampicillin and chloramphenicol as templates and methacrylic acid and 4-vinylpyridine as functional monomers. The dual-template imprinted polymer was used as the capture unit for selective enrichment. When target molecules occupied the imprinted cavities and were drawn into the capillary, they migrated to the hot spots of AgNPs, significantly amplifying the Raman signal. LODs of 1.3 × 10^−7^ M and 1.8 × 10^−7^ M were achieved for ampicillin and chloramphenicol, respectively. The sensor was successfully applied to chicken and milk samples, with recoveries of 97.1–101.5% for ampicillin and 97.7–103.8% for chloramphenicol. In a different approach to complex-matrix tolerance, an Ag/poly(N-isopropylacrylamide)-laponite (Ag/PNIP-LAP) hydrogel membrane was fabricated by UV-induced in situ polymerization, encapsulating AgNPs within a thermoresponsive hydrogel network [126]. The membrane exhibited a molecular sieving effect: hydrophilic small molecules entered the swollen hydrogel network, while hydrophobic macromolecules were excluded (Figure 5A). Upon drying, the hydrogel shrank by up to 10-fold, drawing AgNPs closer to form dense hot spots and concentrating the analytes in confined spaces. This all-in-one separation, enrichment, and detection platform was applied to urotropine in dried bean curd sticks, 2,5-dimethylpyrazine in nuts and potato chips, and pyrazinamide in human plasma, with LODs of 17.4, 31.0, and 53.1 μg/L, respectively, and recoveries ranging from 81.8% to 116.8%. While the inclusion of pyrazinamide in human plasma represents a clinical application outside the primary scope of food safety, this example is retained because the core technology is conceptually valuable for food safety monitoring. The same membrane design can be directly adapted to exclude food matrix interferents while enriching target food contaminants. Both the dual-template sensor and the hydrogel membrane represent multi-target detection, where the same platform is applied to different analytes across separate measurements, relying on selective recognition or matrix separation rather than concurrent spectral identification.

Multi-class spectral classification constitutes the second dimension of multiplexed analysis, where machine learning or chemometric models transform complex SERS spectra into discriminative features for accurate identification and quantification, regardless of whether the targets are present in the same measurement. A flexible three-dimensional hybrid SERS substrate was fabricated by decorating AuNPs onto polypyrrole (PPy) nanospheres, which were subsequently integrated onto a PVDF membrane through vacuum filtration [127]. The PPy@Au-PVDF substrate achieved an enhancement factor of ~10^9^ and an LOD of 10^−11^ M for thiram, which was validated through real-world testing on fruit surfaces. To enable intelligent spectral analysis, an artificial neural network (ANN) model was developed and trained on SERS spectra of thiram, methylene blue, acid orange, indigo carmine, and rhodamine B. The trained model exhibited 100% classification accuracy, effectively distinguishing thiram residues from structurally similar organic pollutants. This platform represents multi-class spectral classification, as the ANN model classifies different analytes based on spectral pattern recognition rather than relying solely on characteristic peak assignment.

In another algorithm-driven strategy, a flexible porous agarose/chitosan substrate was constructed by loading AgNPs through interfacial self-assembly, and the platform was applied to label-free detection of multiple pathogenic bacteria [128]. Detection limits of 8.32, 50.6, 20.8, and 21.2 CFU/mL were reached for *S*. *aureus*, *E*. *coli*, *Salmonella enterica* (*S*. *enterica*), and *Pseudomonas aeruginosa* (*P. aeruginosa*), respectively (Figure 5B). A one-dimensional convolutional neural network (1D-CNN) was employed to analyze the acquired SERS spectra. The 1D-CNN model achieved 100% classification accuracy and accurate concentration prediction for both single and mixed bacterial samples. In real samples including chicken, milk, and pond water, recovery rates ranging from 93% to 118% were obtained. A further advance in multiplexed detection was demonstrated through a flexible substrate that combined plasmonic nanoengineering with chemometric analysis for pesticide residues in complex liquid matrices (Figure 5C) [153]. The substrate was applied to milk samples, where multiple pesticide contaminants were simultaneously determined. Through the integration of spectral preprocessing and multivariate calibration, accurate quantification was achieved even in the presence of strong matrix interference from proteins and lipids. The method exhibited satisfactory recovery rates and RSDs, confirming its reliability for multi-analyte pesticide screening in real dairy products. The combination of spectral preprocessing and multivariate calibration effectively mitigates matrix interference from proteins and lipids, enabling direct analysis of milk without extensive sample cleanup. Both the agarose/chitosan substrate and the pesticide screening platform demonstrate multi-class spectral classification, where machine learning or chemometric models enable accurate identification and quantification in complex matrices.

Building on the integration of machine learning with flexible SERS platforms, a stretchable and breathable hydrogel-elastomer composite tape was developed for in vivo fruit safety surveillance (Figure 5D) [129]. The SERS-active functional elastomeric tape was fabricated by embedding Au-decorated Ag nanowires into an ultrathin hydrogel-elastomer matrix. The tape formed conformal, adhesive contact with moist plant surfaces and enabled bottom-up analyte absorption for direct top-layer SERS readout without substrate inversion. For multiplex detection, thiram and kinetin were simultaneously quantified on mandarin leaves. A support vector regression model was trained using exclusive characteristic peaks of each analyte, achieving *R*^2^ values of 0.99 for kinetin and 0.91 for thiram across a concentration range of 1 μM to 1 mM. The tape also enabled early detection of *Penicillium* spp. infection up to two days before visible symptoms. This platform combines multi-target detection of thiram and kinetin with multi-class spectral classification via support vector regression, illustrating the complementary nature of the two analytical capabilities.

## 4. Challenges and Outlook

Despite significant progress in flexible SERS substrate design and laboratory validation, several key obstacles remain before these platforms can be routinely deployed for on-site food safety monitoring. The transition from laboratory prototypes to field-ready devices requires overcoming challenges in instrument portability, sample preparation simplicity, data analysis accessibility, and scalable manufacturing. Issues such as environmental robustness, operator skill dependence, real-time data processing, and batch-to-batch reproducibility must be systematically addressed. This section discusses the current status and future perspectives of flexible SERS technology in terms of portable spectrometer integration, automated sample handling, intelligent data analysis, and large-scale production.

The transition of flexible SERS substrates from laboratory settings to real-world applications requires seamless integration with portable or handheld Raman spectrometers [154]. Compact optical systems and specially designed fiber-optic probes have been developed to enable efficient laser delivery and signal collection on irregular food surfaces [155]. In parallel, SERS-based hydrogel swabs have been combined with portable readers, allowing rapid on-site screening without complex liquid handling [156]. Regarding the challenge of environmental robustness, since field conditions involve fluctuating temperature, humidity, and mechanical vibration, recent studies have shown that proper encapsulation maintains signal reliability by protecting plasmonic nanostructures from oxidation and physical damage. However, encapsulation layers may reduce analyte accessibility to hot spots, and the long-term stability under extreme temperature variations remains to be validated. Furthermore, dual-modal or multi-modal detection strategies have been explored, integrating SERS with colorimetric, fluorescent, or electrochemical readouts on the same portable platform [157,158,159]. This approach directly tackles the limitation of single-mode SERS in complex matrices by providing cross-validation of results, thereby expanding the detectable analyte range and enhancing overall reliability of on-site food safety monitoring [160,161]. Nevertheless, the increased system complexity and higher cost of multi-modal devices pose practical barriers to widespread adoption.

Complementary to the adaptation of portable Raman instruments, simplification and automation of sample pretreatment serve as another core bottleneck for translating flexible SERS from benchtop to field. To address the challenge of simplifying sample pretreatment and reducing manual steps, microfluidic integration has emerged as a key route to streamline workflows: an electrochemically self-assembled AuNP/indium tin oxide chip was coupled with diazotization to enable one-step derivatization and detection of nitrite in complex food samples, removing the need for multi-step cleanup procedures [162,163,164]. This integration reduces the total analysis time from hours to minutes and lowers the risk of human error. However, microfluidic devices require precise fluid control and may face clogging issues when dealing with particulate-rich food samples. Meanwhile, lightweight paper-based devices support one-step extraction-detection: an Au@AgNRs modified filter paper can directly adhere to fruit and vegetable peels for non-destructive paste-and-read sampling of non-systemic pesticides [39], and an AgNWs@ZIF-8 composite filter paper achieves rapid enrichment and detection of thiram on nectarine surfaces via simple press-based microextraction [165]. These strategies greatly shorten on-site detection cycles and lower dependence on specialized personnel, forming a critical pillar for the practical deployment of flexible SERS in food safety monitoring. A key trade-off remains: simplified sample handling often compromises extraction efficiency compared with laboratory-scale pretreatment, potentially affecting LODs for trace-level contaminants.

Beyond hardware integration and sample preparation, intelligent data analysis and real-time feedback are essential for translating flexible SERS platforms into practical on-site tools [166]. To address the challenge of establishing reliable reference databases for rapid spectral matching, cloud-based spectral databases have been established to store reference spectra of common food contaminants, enabling rapid library matching for unknown sample identification [167]. While cloud-based approaches provide comprehensive reference libraries, they depend on internet connectivity, which is often unavailable in field settings. Edge computing algorithms, deployed directly on portable Raman devices, allow real-time spectral preprocessing and analysis without relying on internet connectivity, which is critical for field operations in remote areas [168,169]. Smartphone applications have been developed to provide intuitive quantitative outputs, converting raw SERS spectra into color-coded maps or numerical concentration readouts through pre-trained calibration models. For instance, a reduced graphene oxide membrane combined with random forest regression achieved a coefficient of determination of 0.9852 for sulfasalazine quantification, and the prediction results were displayed directly on a smartphone interface [170]. Despite these advances, the generalizability of machine learning models remains a concern because models trained on laboratory-collected spectra may perform poorly on field data due to variations in substrate quality, environmental conditions, and sample matrix effects. Expanding training datasets and developing adaptive algorithms will be necessary for robust real-world performance. This integration of cloud-edge-device architecture, machine learning models, and mobile visualization significantly lowers the technical barrier for non-expert users and accelerates decision-making in food safety emergencies.

Large-scale production is a prerequisite for the practical deployment of flexible SERS substrates in routine food safety monitoring. To address the challenges of manufacturing throughput and batch-to-batch reproducibility, several scalable fabrication methods have been developed, including vacuum filtration, roll-to-roll printing, and plasma reduction. Vacuum filtration enables rapid assembly of plasmonic nanoparticles onto flexible membranes with controllable density and uniformity [171]. Roll-to-roll printing offers continuous, high-throughput manufacturing of SERS-active films on polymer substrates, significantly reducing production time and labor costs [172]. Plasma reduction provides a dry, solvent-free route for in situ generation of metallic nanoparticles on various flexible supports [173]. Batch-to-batch reproducibility has been evaluated through relative standard deviations of SERS intensities across multiple independently fabricated substrates [174]. While this level of variability may be acceptable for semi-quantitative screening, it remains insufficient for regulatory-grade quantitative analysis. Future efforts should focus on establishing standardized fabrication protocols, inline quality control mechanisms, and reference materials for performance calibration. These advances in scalable manufacturing, cost control, and quality assurance are essential for transitioning flexible SERS technology from academic research to commercial food safety applications.

Finally, it is important to recognize that the terms “on-site” and “field-deployable” encompass studies with substantially different levels of practical validation. Broadly, three levels can be distinguished: laboratory proof-of-concept using benchtop Raman spectrometers and spiked samples, portable on-surface measurements using handheld Raman instruments on spiked samples, and genuine field validation involving naturally contaminated real samples under actual environments. Most studies reviewed currently fall into the first two levels, with only a few reaching genuine field validation. Beyond substrate flexibility, advancing toward genuine field validation requires system-level integration, including standardized assay formats, automated Raman positioning and acquisition, and high-throughput multiplexed analysis. Recent integrated SERS workflows for foodborne pathogen detection demonstrate how substrate engineering, instrumentation, and data analytics can be unified into complete analytical systems [175,176]. Clearly distinguishing these validation levels will help guide future research toward truly field-ready food safety monitoring technologies.

## 5. Conclusions

Flexible SERS substrates have emerged as a significant technology in the field of food safety monitoring, offering exceptional sensitivity and portability for on-site analysis. This review systematically summarized the evolution of flexible SERS platforms, encompassing rational material selection, innovative structural engineering of supports, and advanced performance enhancement strategies. We highlighted their critical applications in real-world scenarios, particularly in swabbing-based detection on irregular surfaces, in situ analysis of complex food matrices, enrichment-assisted trace analysis, and multiplexed contaminant identification.

Despite significant strides in laboratory settings, the translation of flexible SERS from benchtop to field deployment still faces notable challenges. Key barriers include the seamless integration with portable spectrometers, the simplification of complex sample pretreatment, the establishment of robust cloud-edge-device data ecosystems, and the development of cost-effective, large-scale manufacturing processes. Addressing these issues, such as improving environmental durability, automating microfluidic workflows, and ensuring batch-to-batch reproducibility, is paramount for practical implementation. Looking ahead, the convergence of flexible SERS technology with artificial intelligence, microfluidics, and automated manufacturing heralds a new era for next-generation food safety diagnostics. By overcoming the outlined limitations, flexible SERS substrates are poised to revolutionize real-time food quality control, ensuring enhanced public health protection and fostering greater consumer confidence in the global food supply chain. Future research should focus on standardizing protocols and deepening interdisciplinary collaborations to accelerate the commercialization of this promising technology.

## Figures and Tables

**Table 1:** Representative applications of flexible SERS substrates in food safety monitoring.

Target Analyte and Food Matrix	SERS Substrates	Detection Mode	LOD	Raman Instrumentation	Validation Level	References
Crystal violet on fish skin	Au nanospheres/PS/PDMS	Direct swabbing	0.028 ppm	Benchtop	Spiked food	[110]
Malachite green on Pacific saury skin	Ag@polyamide fabric	Moistened wiping	7.8 × 10^−8^ M	Benchtop	Spiked food	[111]
Crystal violet and malachite green on fish skin	Etched AgNWs/sandpaper-templated PDMS	Press-and-peel swabbing	89 nM, 54 nM	Benchtop	Spiked food	[112]
Fipronil on tomato and chili exocarps	AgNPCs/Mo_2_C@adhesive Al tape	Paste-press-peel swabbing	1.65 × 10^−10^ M, 1.72 × 10^−10^ M	Portable	Spiked food	[113]
Thiram on apple surface	AgNPs@filter paper	Direct wiping	0.005 ng/cm^2^	Portable	Spiked food	[114]
Ciprofloxacin and ampicillin on chicken wing skin	Gold nanostars/adhesive tape	Press-on peel-off swabbing	3.3 nM, 1.5 nM	Portable	Spiked food	[115]
Malachite green on fish skin	AgNPs on Fe_3_O_4_ NPs	Spray-collect	10^−12^ M	Portable	Spiked food	[116]
Methylene blue on crucian carp skin	AgNPs/Al/pyramid PET	In situ detection	10^−6^ M	Benchtop	Spiked food	[117]
Thiram on apple and chili pepper surfaces	CNF/GNR@Ag membrane with hole-punched PDMS	In situ detection	10^−11^ M	Portable	Spiked food	[118]
Thiram on apple peels	Sodium alginate–chitosan @AgNCs freeze-dried gel	In situ detection	0.055 mg/L	Portable	Spiked food	[119]
Thiram on strawberries, apples, mushrooms	Au@AgNR arrays on silicone membrane	In situ detection	2 ng/cm^2^	Portable	Spiked food	[120]
Thiram on apple	Dendritic Ag nanostructures on PDMS	In situ detection	10^−6^ M	Portable	Spiked food	[121]
Acetamiprid in peach and cowpea	Fe_3_O_4_@ZIF-8@Ag	Electro-driven adsorption, magnetic separation	4 nM	Benchtop	Spiked food, real sample	[122]
*E*. *coli* in apple juice	PET/ITO/Ag	Dielectrophoretic concentration	~10^6^ CFU/mL (qualitative detection, no LOD reported)	Benchtop	Spiked food	[123]
Dimethoate on olive surfaces	AuNPs/polyethylene glycol diacrylate hydrogel	Hydrogel swelling enrichment	3.13 ppb	Portable	Spiked food	[124]
Enrofloxacin in milk	Ag nanoflowers/PVA hydrogel	Molecular sieving	47.96 ppb	Benchtop	Spiked food	[125]
Urotropine in dried bean curd sticks; 2,5-dimethylpyrazine in nuts and potato chips	Ag/poly(N-isopropylacrylamide)-laponite hydrogel membrane	Multi-target detection	17.4 μg/L, 31.0 μg/L	Portable	Spiked food	[126]
Thiram, methylene blue, acid orange, indigo carmine, rhodamine B on fruit surfaces	Polypyrrole@Au-PVDF membrane (vacuum filtration)	Multi-class spectral classification	10^−11^ M	Benchtop	Spiked food	[127]
*S. aureus*, *E. coli*, *S. enterica*, *P. aeruginosa* in chicken, milk	AgNPs/agarose-chitosan porous substrate	Multi-class spectral classification	8.32 CFU/mL, 50.6 CFU/mL, 20.8 CFU/mL, 21.2 CFU/mL	Benchtop	Real sample	[128]
Thiram and kinetin on mandarin leaves	Au@Ag NWs/hydrogel-elastomer composite tape	Multi-target detection, multi-class spectral classification	1 nM, 1 μM	Benchtop, Portable	Real sample	[129]

## Data Availability

No new data were created or analyzed in this study. Data sharing is not applicable to this article.
